# Development of a human in vitro blood–brain tumor barrier model of diffuse intrinsic pontine glioma to better understand the chemoresistance

**DOI:** 10.1186/s12987-020-00198-0

**Published:** 2020-06-02

**Authors:** Clémence Deligne, Johan Hachani, Sophie Duban-Deweer, Samuel Meignan, Pierre Leblond, Angel M. Carcaboso, Yasuteru Sano, Fumitaka Shimizu, Takashi Kanda, Fabien Gosselet, Marie-Pierre Dehouck, Caroline Mysiorek

**Affiliations:** 1 Laboratoire de la Barrière Hémato-Encéphalique (LBHE), Univ. Artois, UR 2465, 62300 Lens, France; 2 Laboratoire de la Barrière Hémato-Encéphalique (LBHE), Plateau Spectrométrie de Masse de l’ARTois (SMART), Univ. Artois, UR 2465, 62300 Lens, France; 3 Institut National de la Santé et de la Recherche Médicale (INSERM), U908, 59000 Lille, France; 4 Institut pour la Recherche sur le Cancer de Lille (IRCL), 59000 Lille, France; 5 Unité Tumorigenèse et Résistance aux Traitements, Centre Oscar Lambret, 3 rue Frédéric Combemale, 59000 Lille, France; 6 Département de Cancérologie pédiatrique, Institut d’Hématologie et d’Oncologie Pédiatrique, 69000 Lyon, France; 7 Institut de Recerca Sant Joan de Deu, Esplugues de Llobregat, 08950 Barcelona, Spain; 8 Department of Neurology and Clinical Neuroscience, Yamaguchi University Graduate School of Medicine, Ube, Japan

**Keywords:** Blood–brain barrier, Blood–brain tumor barrier, Diffuse intrinsic pontine glioma, Chemoresistance, Pediatric brain cancer

## Abstract

**Background:**

Pediatric diffuse intrinsic pontine glioma (DIPG) represents one of the most devastating and lethal brain tumors in children with a median survival of 12 months. The high mortality rate can be explained by the ineligibility of patients to surgical resection due to the diffuse growth pattern and midline localization of the tumor. While the therapeutic strategies are unfortunately palliative, the blood–brain barrier (BBB) is suspected to be responsible for the treatment inefficiency. Located at the brain capillary endothelial cells (ECs), the BBB has specific properties to tightly control and restrict the access of molecules to the brain parenchyma including chemotherapeutic compounds. However, these BBB specific properties can be modified in a pathological environment, thus modulating brain exposure to therapeutic drugs. Hence, this study aimed at developing a syngeneic human blood–brain tumor barrier model to understand how the presence of DIPG impacts the structure and function of brain capillary ECs.

**Methods:**

A human syngeneic in vitro BBB model consisting of a triple culture of human (ECs) (differentiated from CD34^+^-stem cells), pericytes and astrocytes was developed. Once validated in terms of BBB phenotype, this model was adapted to develop a blood–brain tumor barrier (BBTB) model specific to pediatric DIPG by replacing the astrocytes by DIPG-007, -013 and -014 cells. The physical and metabolic properties of the BBTB ECs were analyzed and compared to the BBB ECs. The permeability of both models to chemotherapeutic compounds was evaluated.

**Results:**

In line with clinical observation, the integrity of the BBTB ECs remained intact until 7 days of incubation. Both transcriptional expression and activity of efflux transporters were not strongly modified by the presence of DIPG. The permeability of ECs to the chemotherapeutic drugs temozolomide and panobinostat was not affected by the DIPG environment.

**Conclusions:**

This original human BBTB model allows a better understanding of the influence of DIPG on the BBTB ECs phenotype. Our data reveal that the chemoresistance described for DIPG does not come from the development of a “super BBB”. These results, validated by the absence of modification of drug transport through the BBTB ECs, point out the importance of understanding the implication of the different protagonists in the pathology to have a chance to significantly improve treatment efficiency.

## Background

Brain tumors represent the leading cause of cancer-related death in children. Among them, 80% of pediatric brain stem tumors arise in the pons [1]. Diffuse intrinsic pontine glioma (DIPG) are grade IV tumors associated with a high mortality rate and a median survival that is less than 1 year from the day of diagnosis [2]. This high mortality rate is linked to the inefficiency of current therapeutic approaches. Indeed, patients with DIPG are not eligible for surgical resection due to the anatomic location and the diffuse pattern of the tumor. Moreover, as chemotherapy has failed to show any benefit [3], the standard of care remains focal radiotherapy [4], which provides only temporary relief [5]. Despite the numerous clinical trials held on high-grade glioma (HGG), many of them were inconclusive because of the persistent assumption that pediatric HGGs are similar to their adult counterparts. HGGs represent a heterogeneous class of tumors. Recently, the new World Health Organization (WHO) classification of brain tumors based on recent molecular data highlighted the fact that pediatric HGGs are markedly different from their adult counterpart in terms of localization and molecular characteristics. This indicates that the findings concerning adult HGGs cannot be simply extrapolated to pediatric patients and that there is a need for a specific research dedicated to DIPG patients [2, 6]. Hence, these tumors remain incurable mainly due to their chemoresistance and the lack of relevant representative models allowing the understanding of pathological mechanisms and the testing of new therapeutics. For a few years, biopsies have been more widely practiced in DIPG patients [4], rendering human tumor tissue more accessible for the development of in vivo rodent models, such as xenograft models and genetically engineered mouse models (GEMMs). In vivo approaches are useful to study the oncogenesis of the tumor and the overall response to treatments, nonetheless they present some limitations. On one hand, xenograft models performed on immunodeficient mice have the disadvantage of not considering the immune system in the pathogenesis [7]. On the other hand, GEMMs, present the advantage of having clearly identified genetic alterations and recapitulate the complete de novo development of the tumor with the right kinetic and brain location, thus giving some useful information relative to the vascular development around the tumor. However, the complete de novo development of brain tumors in mice can induce a host adaptation leading to the wrong identification of the molecular protagonists involved in the pathogenesis [8]. For these reasons, the use of animal models render difficult the translation of experimental results to the clinic since species differences are observed and have an impact on the prediction of drug delivery into the brain [8, 9].

In addition to the in vivo approaches, human cellular models are important to study cellular and molecular mechanisms. DIPG cells are used in vitro to clarify their response to drugs and to evaluate the efficiency of new treatments. Chemoresistance, which is a major problem in the treatment of cancer, is observed in DIPG patients. However, surprisingly, DIPG cells were demonstrated to be chemosensitive in a recent in vitro*in vitro* study, highlighting that these cancer cells are not chemoresistant per se [10]. Veringa et al. suggest the involvement of the blood–brain barrier (BBB) in the drug resistance phenomenon by restricting the ability of drugs to reach the cancer cells [10].

The BBB represents the main entry to the central nervous system (CNS). Localized at the brain capillaries, the BBB has a specific architecture where endothelial cells share a common basement membrane with pericytes and the overall capillaries are covered by astrocyte endfeet. Pericytes and astrocytes play a critical role in the development and the maintenance of the BBB [11–14]. Neurons directly connected to the brain capillaries and microglial cells also take part in the modulation of the BBB function in physiological and pathological conditions [15]. The BBB has specific properties to control and restrict access to the CNS in order to maintain brain homeostasis. The BBB ECs represent a physical barrier with the establishment at the paracellular level of a complex of tight junction proteins (claudins, occludin, zonula-occludens..) which seals the intercellular spaces. The crossing of the BBB ECs is also restricted via the transcellular way by the metabolic barrier properties, consisting of the efflux pump system [16] and drug metabolizing enzymes, including detoxification enzymes (e.g. monoamine oxidase, cytochrome P450) described in many organs and also present at the BBB. Consequently, these selective properties represent a protection for brain cells against neurotoxic compounds but also an obstacle to overcome for most therapeutic drugs to reach the brain parenchyma at an efficient dose [12, 16–18]. Indeed, the cytochrome P450 (CYP) enzymes are involved in the metabolism of many endogenous (e.g. sterols, vitamins…) and exogenous substances [19], and work together with efflux transporters to limit the entry of drugs to the brain [20].

The BBB has a dynamic regulation of its properties through the communications with the surrounding cells. In the case of a brain tumor, the new environment interferes with these communications and induces modifications of the physical and metabolic properties of the BBB, which is then renamed blood–brain tumor barrier (BBTB) [12, 21]. There is a need for understanding the behavior of the BBTB in the DIPG environment, which could modify the tumor exposure to chemotherapeutic drugs and consequently modulate treatment efficiency. Although the maintenance of the physical BBB/BBTB integrity is well characterized in DIPG patients, little is known about its properties at the cellular and molecular levels [22]. Hence, the aim of the study is to characterize the physical and metabolic properties of the BBTB to clarify its role in the chemoresistance observed in DIPG patients. To do so, a human syngeneic in vitro BBTB model specific to the DIPG was developed. Moreover, to determine the impact of the DIPG environment on the transport of chemotherapeutic drugs, the permeability of the BBTB ECs to chemotherapeutic compounds was measured. The results presented in this study help to clarify the involvement of the BBTB in the chemoresistance described in DIPG patients, and provide an original and useful model for further studies of new therapeutic candidates in the field of pediatric neurooncology.

## Methods

### Co-culture model of the BBB

#### Culture of human endothelial cells

Endothelial cells (ECs) were derived from CD34^+^ hematopoietic stem cells isolated from human umbilical cord blood according to the method described by Pedroso et al. [23]. Written and informed consent from the donor’s parents was obtained for the collection of umbilical cord blood, in compliance with the French Legislation. The protocol was approved by the French Ministry of Higher Education and Research (reference: CODECOH DC2011-1321) and by the local investigational review board (Béthune Maternity Hospital, Beuvry, France). Once isolated from umbilical cord blood, CD34^+^-cells were differentiated in vitro into ECs using endothelial cell growth medium (EGM; Lonza Walkersville, MD, USA) containing 50 ng/mL vascular endothelial growth factor (PeproTech, Rocky Hill, USA) and 20% heat-inactivated fetal calf serum (FCS; Sigma Aldrich, St Louis, MO, USA).

#### Culture of human pericytes

Human brain pericytes were provided by Professor Takashi Kanda (Department of Neurology and Clinical Neuroscience, Yamaguchi University Graduate School of Medicine, Ube, Japan). As described previously by Kanda’s team [24], pericytes were isolated from human brain tissue and immortalized via transfection with retrovirus vectors incorporating human temperature-sensitive SV40 T antigen (tsA58) and human telomerase (hTERT). Cells were grown in Dulbecco’s modified Eagles’ medium containing 4.5 g/L d-glucose, supplemented with 10% heat-inactivated FCS, 1% l-glutamine (Merck Chemicals, Darmstadt, Germany) and 1% penicillin–streptomycin (Sciencell, Carlsbad, CA, USA).

#### Co-culture experimental design

Our syngeneic BBB in vitro model is based on the co-culture of human CD34^+^-derived ECs with the human brain pericytes, instead of the initial bovine ones [25]. To perform the syngeneic contact co-culture model, Transwell inserts (12-well, 0.4 µm; Corning Inc., New York, NY, USA) were flipped over and coated with rat tail type I collagen (10 µg/cm^2^; Corning). After an enzymatic dissociation using trypsin, 4.46 × 10^4^ pericytes/cm^2^ were seeded on the lower surface of each insert and placed 3 h at 37 °C. The inserts were flipped over again to be placed in a 12-well plate and coated with Matrigel™ (BD Biosciences, San Jose, CA, USA) on their upper surface. Subsequently, CD34^+^-derived ECs were seeded on the inserts (7.14 × 10^4^ cells/cm^2^). The model was cultured in a humidified 5% CO_2_ atmosphere in ECM-5 medium, which consists of basal endothelial cell medium (ECM; Sciencell) supplemented with 5% heat-inactivated FCS, 1% endothelial cell growth supplement (Sciencell, Carlsbad, CA, USA) and 0.5% gentamicin (Biochrom AG, Berlin, Germany). The medium was renewed every 2 days. After 6 days of co-culture, ECs acquired the BBB phenotype and were used for experiments.

### Triple culture model of the BBTB and the BBB

#### Culture of human DIPG cells

Patient-derived cells were obtained under an Institutional Review Board-approved protocol and with written informed consent (M-1608-C) at Hospital Sant Joan de Deu Barcelona, Spain. The cells were kindly provided by Dr. Angel Montero Carcaboso (Institut de Recerca Sant Joan de Deu, Barcelona, Spain). As indicated in Table 1, the cells were isolated either from patient autopsy (HSJD-DIPG-007) [26]; or from biopsies at diagnosis (HSJD-DIPG-013, HSJD-DIPG-014).

**Table 1:** Clinical and experimental characteristics of DIPG cells

Cells	WHO grade	Histology	Age at diagnosis (years)	Sex	Tissue origin	ACVR1 mutation	H3F3A mutation
HSJD-DIPG-007	IV	Glioblastoma multiforme	9.9	M	Autopsy	Mutated	p.Lys27Met or K27M
HSJD-DIPG-013	IV	Anaplastic astrocytoma	6.0	F	Biopsy	WT	p.Lys27Met or K27M
HSJD-DIPG-014	IV	Anaplastic astrocytoma	8.2	F	Biopsy	WT	p.Lys27Met or K27M

*M* male, *F* female, *WT* wild-type

Cells were cultured as tumor spheres in modified Tumor Stem Medium, consisting of 95% DMEM/F12 and Neurobasal-A medium (1:1), 1% HEPES 1 M, 1% MEM Non-Essential Amino Acids, 1% 100 mM Sodium Pyruvate, 1% GlutaMAX and 1% Antibiotic–Antimycotic (all components from Life Technologies, Paisley, UK), supplemented with 2% B27 without vitamin A (Life Technologies), 20 ng/mL human EGF, 20 ng/mL human bFGF, 10 ng/mL human PDGF-AA, 10 ng/mL human PDGF-BB (all growth factors from PeproTech) and 2 µg/mL heparin (Sigma-Aldrich).

All cells were routinely subjected to mycoplasma testing (MycoAlert™, Lonza) and only used for experiments when confirmed negative.

#### Culture of human astrocytes

Astrocytes isolated from human brainstem (HA-bs; Sciencell) were cultured in basal astrocyte medium supplemented with 2% FCS, 1% astrocyte growth supplement and 1% penicillin–streptomycin (all components from Sciencell).

#### Triple culture experimental design

On the fifth day of co-culture between ECs and pericytes, astrocytes and DIPG cells were seeded on 12-well plates (9.47 × 10^4^ cells/cm^2^) in ECM-5 medium. On the sixth day, i.e. when ECs acquired the BBB phenotype, inserts containing ECs and pericytes were transferred above the astrocytes for the BBB model or above the DIPG cells for the BBTB model, for an incubation period of 24 h, 72 h or 7 days. Except for the incubation period of 24 h, the medium was changed every 2 days.

### Permeability assays

#### Permeability to BBB integrity marker

Diffusion of the small hydrophilic molecule Lucifer Yellow (LY; Sigma Aldrich), which poorly crosses the BBB, was measured to assess the ECs physical integrity. The inserts, containing ECs on the luminal side and pericytes on the abluminal side, were transferred into 12-well plates containing 1.5 mL of Ringer-HEPES solution (RH; 150 mM NaCl, 5.2 mM KCl, 2.2 mM CaCl_2_, 0.2 mM MgCl_2_6H_2_O, 6 mM NaHCO_3_, 5 mM HEPES, 2.8 mM glucose; pH 7.4) per well. The insert’s cell culture medium was removed and 0.5 mL of RH solution containing 50 μM LY (excitation/emission wavelengths: 432/538 nm) was added to the luminal compartment. Cells were then placed at 37 °C. After several time points (20, 40 and 60 min), inserts were placed in new wells containing RH solution. An aliquot was taken from the abluminal compartments of each time point of the kinetic, from the luminal compartment of the last time point and from the initial solution for quantification with a fluorimeter (Synergy™ H1, BioTek Instruments, Winooski, VT, USA). The clearance principle was used to obtain a concentration-independent transport parameter. The cleared volume was calculated by dividing the amount of LY in the abluminal compartment by the concentration in the donor compartment. The mean volume cleared was plotted vs time. The slope was estimated by linear regression. The slope of the clearance curve gives the PS*f* (for inserts with the Matrigel™ coating, the collagen I coating and the pericytes) and P*St* (for inserts with the ECs, the Matrigel™ coating, the collagen I coating and the pericytes) values, where PS is the permeability surface area product. The PS value of the ECs monolayer (PS*e*) was calculated by applying the following formula: 1/PS*e* = 1/PS*t *− 1/PS*f*. Finally, to generate the ECs permeability coefficient (Pe, in cm/min), the PSe value was divided by the surface area of the porous membrane of the insert (1.12 cm^2^).

#### Permeability to chemotherapeutic drugs

The ECs Pe values of temozolomide (TMZ) and panobinostat (Selleck Chemicals, Houston, TX, USA) were obtained using the same experimental and calculation methods as the ones described above for LY. Drug solutions were prepared in RH solution at a final concentration of 50 µM for TMZ and 10 µM for panobinostat. All drugs were co-incubated with LY at 50 µM to assess the barrier integrity.

Quantification of the drugs was performed using a LC–MS/MS system consisting of an AB SCIEX TripleTOF^®^ 5600 mass spectrometer (AB Sciex, Singapore) outfitted with an Ekspert™ nanoLC 400 System. Separation was carried out on an Eksigent HALO C18 reverse phase column (0.5 × 50 mm, particle size 2.7 μm) using a linear gradient method. The mobile phase used for the chromatographic separation was composed of 0.1% (v/v) formic acid in water (mobile phase A) and 0.1% (v/v) formic acid in acetonitrile (mobile phase B) with a flow rate of 15 μL/min. The initial mobile phase composition was 60% mobile phase A and 40% mobile phase B. From 0.5 to 4.0 min, mobile phase B was increased linearly from 40 to 80% and maintained until 5.0 min. From 5.0 to 5.1 min, the gradient decreased to 40% mobile phase B and the conditions were maintained until 6 min to re-equilibrate the column for the next injection. The temperature of the column was maintained at 35 °C, whereas the temperature of the autosampler was kept at 8 °C. The injection volume was 10 μL. The column eluent was directed into the AB Sciex TripleTOF^®^ 5600 system then was operated in positive ion mode by Electrospray Ionization using the DuoSpray™ and Turbo V™ ionization sources and controlled by the Analyst^®^ software (version 1.7.1). The Calibrant Delivery System was used for continuous recalibration between every 5 injections. Each chromatography run was approximately ten minutes long. All compounds were analyzed using Multiple Reaction Monitoring (MRM) mode with two transitions per compound. The most sensitive, first MRM transition was used for quantitation while the second MRM transition was used for qualitative identification. The detected MRM transitions with corresponding compound dependent parameters are given in Table 2. The acquired MRM data were processed using PeakView^®^ software (version 2.2) and quantified with the MultiQuant™ software (version 3.0).

**Table 2:** Multiple reaction monitoring (MRM) transitions, compound dependent parameters, and retention times

Substance	Q1	Q3 quantifier	Q3 qualifier	DP	CE	Retention time (min)
Temozolomide	194.15	138.1	177.18	35	13	1.4
Panobinostat	349.43	158.1	309.22	76	22	2.5

*DP* declustering potential, *CE* collision energy, *Q1/Q3* first and third

### Efflux pump functionality

A Rhodamine 123 (R123) accumulation assay was performed to assess the activity of the efflux transporters P-gp and BCRP in ECs. Pericytes were gently scraped off the insert membranes and inserts were transferred in 12-well plates filled with RH solution containing 0.1% BSA. The cell culture medium in the luminal compartments was removed and ECs were incubated with 0.5 mL of R123 (5 µM; Sigma Aldrich) in RH solution supplemented with 0.1% BSA, with or without elacridar (0.5 µM; Sigma Aldrich). After 2 h at 37 °C, the reaction was stopped by rinsing the cells 5 times with ice-cold RH solution. ECs were lysed with RIPA buffer (Merck Millipore, Burlington, MA, USA). The quantification of R123 (excitation/emission wavelengths: 501/538 nm) in samples was carried out using the fluorimeter Synergy™ H1 (BioTek Instruments). The values obtained were normalized by the quantity of proteins.

### Immunocytochemistry

All cell types were fixed in cold methanol/acetone (50%/50%) for 1 min. After three rinses in calcium- and magnesium-free phosphate buffered saline (PBS-CMF; 8 g/L NaCl, 0.2 g/L KCl, 0.2 g/L KH_2_PO_4_, 2.86 g/L NaHPO_4_ − 12 H_2_O; pH 7.4), cells were incubated with the blocking solution consisting of PBS-CMF supplemented with 10% normal goat serum (NGS).

For ECs staining, pericytes were gently scraped off the insert membranes, which were then cut out with a scalpel and incubated with primary antibodies against zonula occludens-1 (ZO-1; rabbit anti ZO-1, ref. 61–7300; Invitrogen, Rockford, IL, USA) and Claudin-5 (rabbit anti-Claudin-5, ref. 34–1600; Invitrogen).

Pericytes were incubated with primary antibodies against platelet-derived growth factor receptor beta (PDGFR-β; rabbit anti-PDGFR-β, ref. ab51090; Abcam, Cambridge, UK), desmin (mouse anti-desmin, ref. ab6322; Abcam) and α-smooth muscle actin (α-SMA; mouse anti-α-SMA, ref. M0851; Dako, Glostrup, Denmark). Astrocytes and DIPG cells were incubated with primary antibody against glial fibrillary acid protein (GFAP; rabbit anti-GFAP, ref. Z0334; Dako). Following three rinses with PBS-CMF supplemented with 2% NGS, cells were incubated with the secondary antibody goat anti-rabbit Alexa Fluor^®^488 (ref. A-11034, Life Technologies, Eugene, OR, USA) or goat anti-mouse Alexa Fluor^®^488 (ref. A-11029, Life Technologies) for 30 min in the dark. All antibodies were diluted in PBS-CMF 2% NGS and used at room temperature (RT). Nuclei were stained using Hoechst reagent. Finally, preparations were mounted using Mowiol (Sigma Aldrich) containing an antifading agent (DABCO, Sigma Aldrich) and observed with a Leica DMRD fluorescence microscope (Leica Microsystems, Wetzlar, Germany). Images were acquired with a high-resolution camera (Cool Snap RS Photometrics; Leica Microsystems).

### RNA extraction and gene expression analysis

After 7 days of incubation with astrocytes or DIPG cells, pericytes were scraped off the abluminal side of the insert membrane, and endothelial cells were rinsed with cold RH buffer. mRNA from endothelial cells was extracted using the NucleoSpin^®^ RNA/protein kit from Macherey-Nagel (MACHEREY-NAGEL, Dueren, Germany). Once isolated, the purity and concentration of mRNA were assessed by measuring the absorbance at 260, 280, and 320 nm using Biotek’s Synergy^TM^ H1 spectrophotometer and Take 3^TM^ plate (BioTek Instruments). For each condition, cDNA was obtained from 250 ng of mRNA using IScript™ Reverse Transcription Supermix (BioRad, Hercules, CA, USA), according to manufacturer’s instructions. qPCR reactions (10µL) were prepared using SsoFast™ EvaGreen^®^ Supermix (BioRad), primers at a final concentration of 100 nM, water and cDNA. The specificity and efficacy of all primer pairs, which are listed in Table 3, were tested before use. qPCR amplification was carried out for 40 cycles with an annealing temperature of 60 °C in a CFX96 thermocycler (BioRad). Ct data were obtained using the Bio-Rad CFX Manager software. Gene expression levels of the targets were calculated relative to the housekeeping gene *PPIA* (Cyclophilin A).

**Table 3:** DNA primers used for the analysis of efflux transporter and CYP enzyme mRNA expression in the endothelial monolayer from the BBB and BBTB models

Target	Gene	Primer F/R	Primer sequence
P-gp	ABCB1	F	CAGACAGCAGCTGACAGTCCAAGAACAGGACT
		R	GCCTGGCAGCTGGAAGACAAATACACAAAATT
BCRP	ABCG2	F	TGGCTGTCATGGCTTGAGTA
		R	GCCACGTGATTCTTCCACAA
MRP1	ABCC1	F	GTCCTTAAACAAGGAGGACACG
		R	TCCTTGGAGGAGTACACAACCT
MRP2	ABCC2	F	CCAATCTACTCTCACTTCAGCGAGA
		R	AGATCCAGCTCAGGTCGGTACC
CYP1A1	CYP1A1	F	TTTTACATCCCCAAGGGGCG
		R	TCTCACCGATACACTTCCGC
CYP1B1	CYP1B1	F	CCACTGAAGTGGCCTAACCC
		R	CCACTGAAGTGGCCTAACCC
CYP2D6	CYP2D6	F	TCATCACCAACCTGTCATCGG
		R	CCTCCGGCTTCACAAAGTGG
CYP2S1	CYP2S1	F	GATCTACAGCCCCTGTTCGG
		R	TTAATTCCCAAGCCGGACCC
CYP2U1	CYP2U1	F	CAGCCATTTGGGAGAAACCG
		R	TGTTCTCCCATACACACCCG
Cyclophilin A	PPIA	F	CTGAGGACTGGAGAGAAAGGAT
		R	GAAGTCACCACCCTGACACATA

### Protein extraction and immunoblotting

After 7 days of incubation with astrocytes or DIPG cells, pericytes were scraped off the abluminal side of the insert membrane. All extraction steps were then performed at 4 °C. Endothelial cells were rinsed twice with RH solution and scraped in 50 µL RIPA lysis buffer (Merck Millipore) supplemented with 3% protease and phosphatase inhibitors (ref. P8340, P5726 and P0044; Sigma Aldrich). After centrifugation of cell lysates for 10 min at 10,000 rpm, supernatants were collected and sonicated twice at 40 W for 5 s. Protein concentration in each sample was determined using the Bradford method.

For each experimental condition, 20 µg of total proteins mixed with Laemmli buffer (Bio-Rad) supplemented with β-mercaptoethanol (Sigma Aldrich) were electrophoresed on Criterion™ TGX™ (Tris–Glycine eXtended) precast gels (Bio-Rad). Proteins were subsequently electrotransferred onto nitrocellulose membranes (GE Healthcare, Little Chalfont, UK). Membranes were incubated for 90 min at RT in Tris-buffered saline 0.1% Tween 20 (TBST; 20 mM Tris–HCl pH 8.0, 500 mM NaCl, 0.1% Tween 20; Bio-Rad) supplemented with 5% skimmed milk. After this blocking step, membranes were incubated with the primary antibody mouse anti-P-gp (ref. C219; GeneTex, Ivine, CA, USA) for 2 h at RT or the primary antibody mouse anti-BCRP (ref. ab3380; Abcam) overnight at 4 °C. For β-actin, the primary antibody mouse anti-β-actin (ref. A5441; Sigma Aldrich) was used at RT for 20 min. Membranes were then rinsed several times with TBST and incubated with the horseradish peroxidase (HRP)-conjugated secondary antibody goat anti-mouse (ref. P0447; Dako) for 1 h at RT. Following another rinsing step, the HRP was assayed using an enhanced chemiluminescence kit (GE Healthcare) and revealed by the Western blot Imaging system Azure c600 (Azure Biosystems, Dublin, Ireland). The bands’ optical densities were measured using the TotalLab TL 100 1D Gel Analysis software (Nonlinear Dynamics, Newcastle, UK). Quantification of the targets’ protein levels was calculated relative to β-actin.

### Statistical analyses

For permeability and transcriptional expression measurements, results were expressed as mean ± SEM. For R123 accumulation assays, results were expressed as percentage ± SEM. Each experiment was performed in triplicate. For analysis involving two groups, an unpaired t-test was used. For analysis of more than two groups, a 1-way ANOVA was used, followed by a Bonferroni’s multiple comparison test. All data were analyzed by the GraphPad Prism 5.0 software (GraphPad Software, San Diego, CA, USA). The threshold for statistical significance was set to p < 0.05.

## Results

### The blood–brain tumor barrier (BBTB) model

**Fig. 1:**
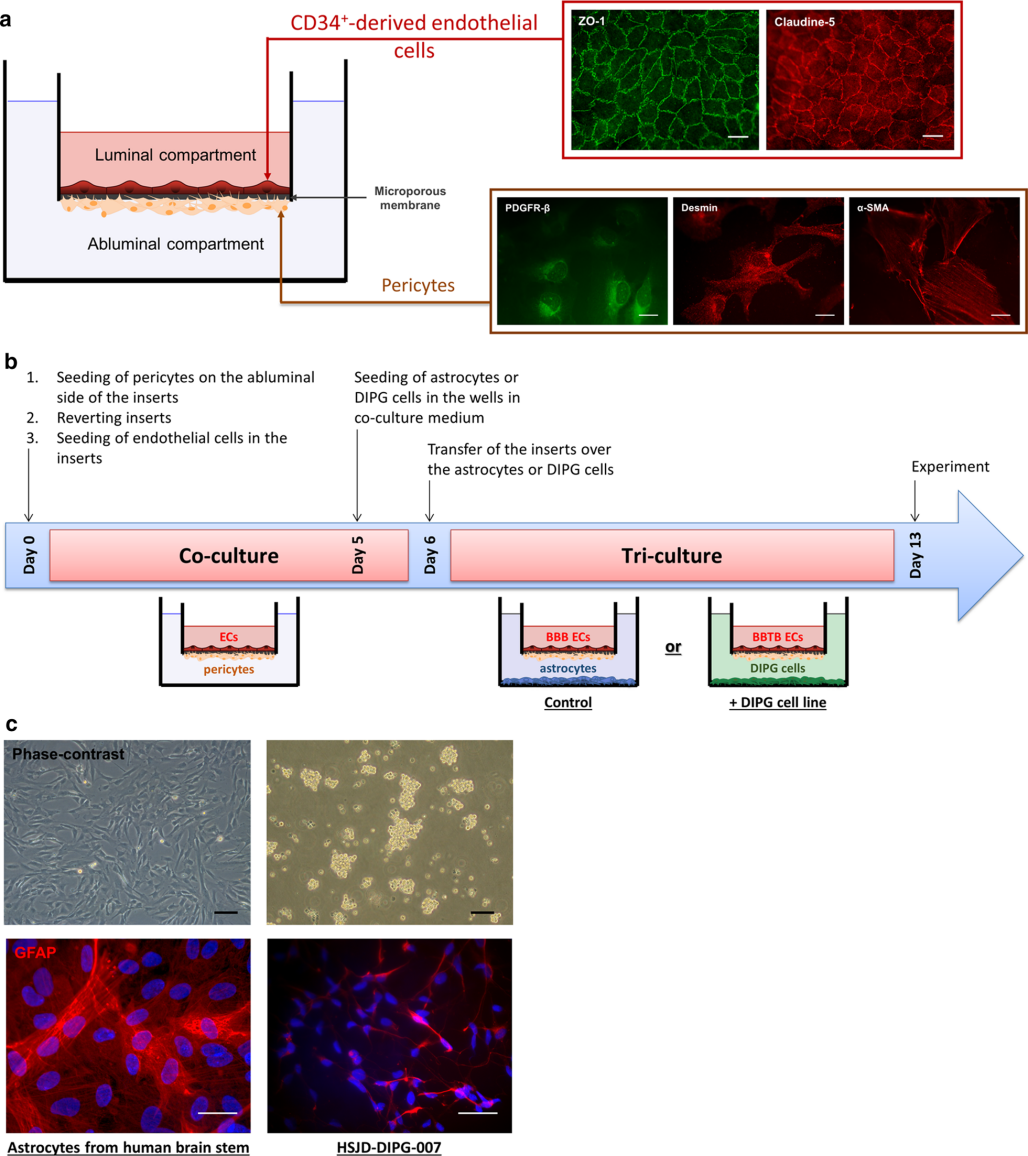
Modelling of the human blood- brain tumor barrier in pediatric DIPG. **a** Shematic representation of the syngeneic human in vitro BBB model where endothelial cells (ECs) were differentiated from human cord blood CD34^+^ hematopoietic stem cells and cultured on the luminal side of the insert. ECs were co-cultured with human brain pericytes, seeded on the abluminal side of the insert. Cells were visualized by performing immunostainings of platelet-derived growth factor receptor-β (PDGFR-β), desmin and α-smooth muscle actin (α-SMA) for pericytes, and tight junction proteins zonula occludens-1 (ZO-1) and claudin-5 for ECs. Scale bar = 25 µm. **b** Synopsis of culture, starting on day 0 with the seeding of ECs and pericytes on the luminal side and the abluminal side of the insert, respectively. After 6 days of co-culture, ECs and pericytes were incubated with either the astrocytes, to model the healthy BBB (control), or the DIPG cells, to model the blood–brain tumor barrier (+ DIPG cell line), for a duration of 7 days. **c** Representative images of glial fibrillary acid protein (GFAP) immunostainings on human astrocytes and DIPG-007 cells. For phase-contrast pictures, scale bar = 50 µm. For immunofluorescence pictures, scale bar = 25 µm

In order to develop the BBTB model, the patented human BBB in vitro model (Brain Like Endothelial Cells model, BLEC) developed in the laboratory and consisting of CD34^+^stem cells-derived endothelial cells (CD34^+^-ECs) co-cultivated with bovine pericytes [25], was adapted. Firstly, human pericytes [27] were used instead of the bovine pericytes in order to have a human syngeneic approach. Moreover, the configuration of the co-culture model was modified to add a third cell type in the culture system. To do so, the human pericytes, characterized by the expression of a set of pericytes markers [28] like platelet-derived growth factor receptor-beta (PDGFR-β), alpha-smooth muscle actin (α-SMA) and desmin, were seeded on the bottom of the insert and the CD34^+^-ECs on the top of the insert (Fig. 1a). The co-culture of the human CD34^+^-ECs with the pericytes aims at inducing the BBB properties in the CD34^+^-ECs. Hence, after 6-days of coculture, the BBB restrictive properties were analyzed in the human BBB endothelial cells, such as the permeability coefficient to Lucifer Yellow (Pe^LY^), a BBB integrity marker, and the immunostaining of the tight junction proteins. After 6 days of co-culture with the human pericytes, the ECs displayed a low Pe^LY^ value (Pe^LY^ = 0.54 ± 0.03 × 10^−3^ cm/min), similar to the value obtained with the original model [25] associated with a continuous localization of the tight junction proteins, ZO-1 and Claudin-5, at the cell junctions (Fig. 1a). In this new configuration, the ECs displayed the same restrictive properties, essential for an in vitro BBB model, as the initial BLEC model [25].

In order to set the BBTB model and study the influence of diffuse intrinsic pontine glioma (DIPG) environment on the ECs properties, at day 6, the inserts containing ECs and pericytes were transferred upon wells containing the pediatric DIPG cancer cell cultures (Fig. 1b). The cancer cell line HSJD- DIPG-007 (DIPG-007) expresses the astrocytes marker, glial fibrillary acid protein (GFAP) and was used to develop the BBTB model (Fig. 1c). As cancer is considered to be a chronic disease, the incubation of the ECs and pericytes in the cancer environment was performed during a kinetic from 24 h to 7 days (Fig. 1b). The ECs were then renamed BBTB ECs.

Moreover, in parallel, a triple culture control model was made by transferring the inserts with ECs and pericytes over wells containing human astrocytes isolated from brainstem, instead of DIPG cells, the ECs were then renamed BBB ECs (Fig. 1b).

The first part of the results set the triple culture models, then the physical and metabolic properties of the BBTB ECs (DIPG pathological condition) were compared to their physiological counterpart, the BBB ECs.

### Physical and metabolic properties of the BBTB

**Fig. 2:**
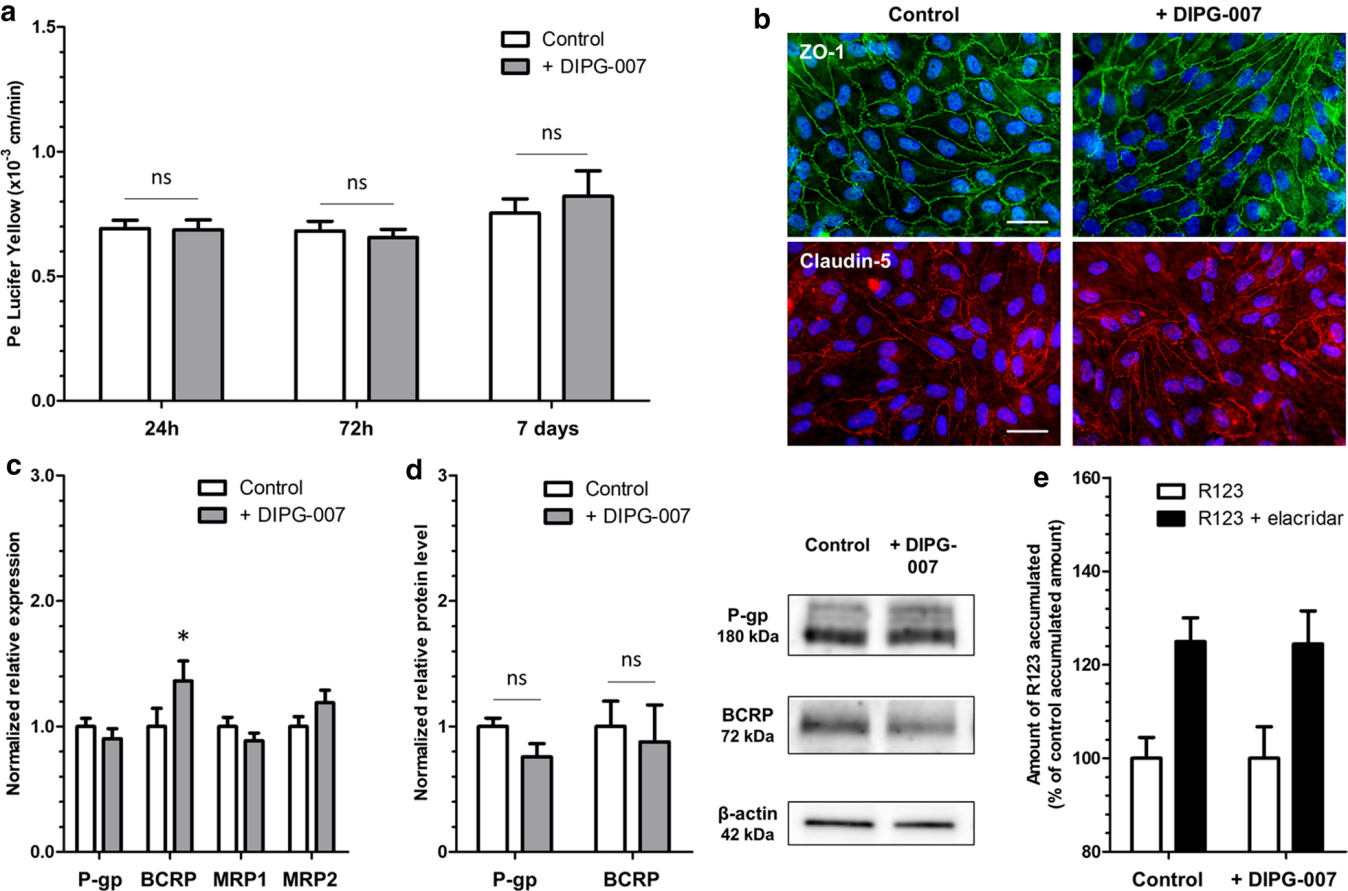
Characterisation of physical and metabolic properties of the blood–brain tumor barrier in DIPG. **a** Endothelial paracellular permeability to Lucifer Yellow after 24 h, 72 h and 7 days of incubation with astrocytes (control) or DIPG-007 cells. P*e* = endothelial permeability coefficient. N = 3; n = 9. ns = non significant. **b** Representative images of tight junction protein immunostainings of zonula occludens-1 (ZO-1) and claudin-5, after 7 days of incubation. Scale bar = 25 µm. **c** Transcriptional expression of the genes of efflux transporters P-gp, BCRP, MRP1, MRP2, after 7 days of incubation, quantified by RT-qPCR and normalized on the expression of the housekeeping gene *PPIA* (cyclophilin A). *p < 0.05. N = 2; n = 6. **d** Protein level of efflux transporters P-gp and BCRP, after 7 days of incubation, quantified by Western blot and normalized on the protein level of β-actin. Results were obtained from two independent experiments (N = 2). ns = non significant. **e** P-gp and BCRP activity, evaluated by the measure of P-gp/BCRP substrate rhodamine 123 (R123) accumulation in ECs, with or without the P-gp/BCRP inhibitor elacridar, after 7 days of incubation. N = 3; n = 9

The impact of the DIPG environment on the physical and the metabolic properties of the BBTB ECs was analyzed and compared to the BBB ECs. First of all, the physical integrity of the BBTB ECs was not compromised compared to the BBB ECs as revealed by the low Pe^LY^ values from 24 h (Pe^LY^ = 0.69 ± 0.03 × 10^−3^ cm/min and 0.68 ± 0.03 × 10^−3^ cm/min respectively) to 7 days (Pe^LY^ = 0.75 ± 0.05 × 10^−3^ cm/min and 0.82 ± 0.10 × 10^−3^ cm/min respectively) (Fig. 2a). This result was correlated with a continuous tight junction staining of ZO-1 and Claudin-5 at the cell borders (Fig. 2b).

The metabolic properties of the BBTB ECs were then evaluated by measuring the expression of efflux transporters from the ATP binding cassette family (ABC family), P-gp, BCRP, MRP1 and MRP2 (genes names *ABCB1, ABCG2*, *ABCC1* and *ABCC2* respectively), quantified by real-time quantitative PCR (Fig. 2c) and western blot for the P-gp and BCRP (Fig. 2d). While a 36% significant increase in the relative expression of *ABCG*2 in the BBTB ECs after 7 days compared to the BBB ECs was measured, no upregulation of the expression of *ABCB1*, *ABCC1* and *ABCC2* was detected (Fig. 2c). The protein levels of P-gp and BCRP were not modified with the DIPG-007 cells compared to control (Fig. 2d).

Moreover, the relationship between mRNA expression, protein level and activity of efflux transporters was investigated. To do so, the intracellular accumulation of Rhodamine 123 (R123), a substrate of P-gp/BCRP, was quantified in the absence or presence of elacridar, an inhibitor of P-gp/BCRP. In the presence of elacridar, the BBB ECs accumulated more R123 reflecting an active efflux transport. The same increase in R123 accumulation was measured in the BBTB ECs compared to the BBB ECs in the presence of elacridar (25% vs 24% respectively) revealing an absence of decrease or increase in P-gp/BCRP activity (Fig. 2e).

Overall, these results showed that the integrity of the BBTB ECs was not compromised under the DIPG-007 environment compared to the BBB ECs, and no modifications of efflux pump mRNA expression, protein level nor activity were measured in the BBTB ECs. In order to validate the BBTB model and analyze if its behavior is reproducible in a DIPG environment, additional DIPG cell cultures were used.

### Additional DIPG cells display the same influence on the BBTB

**Fig. 3:**
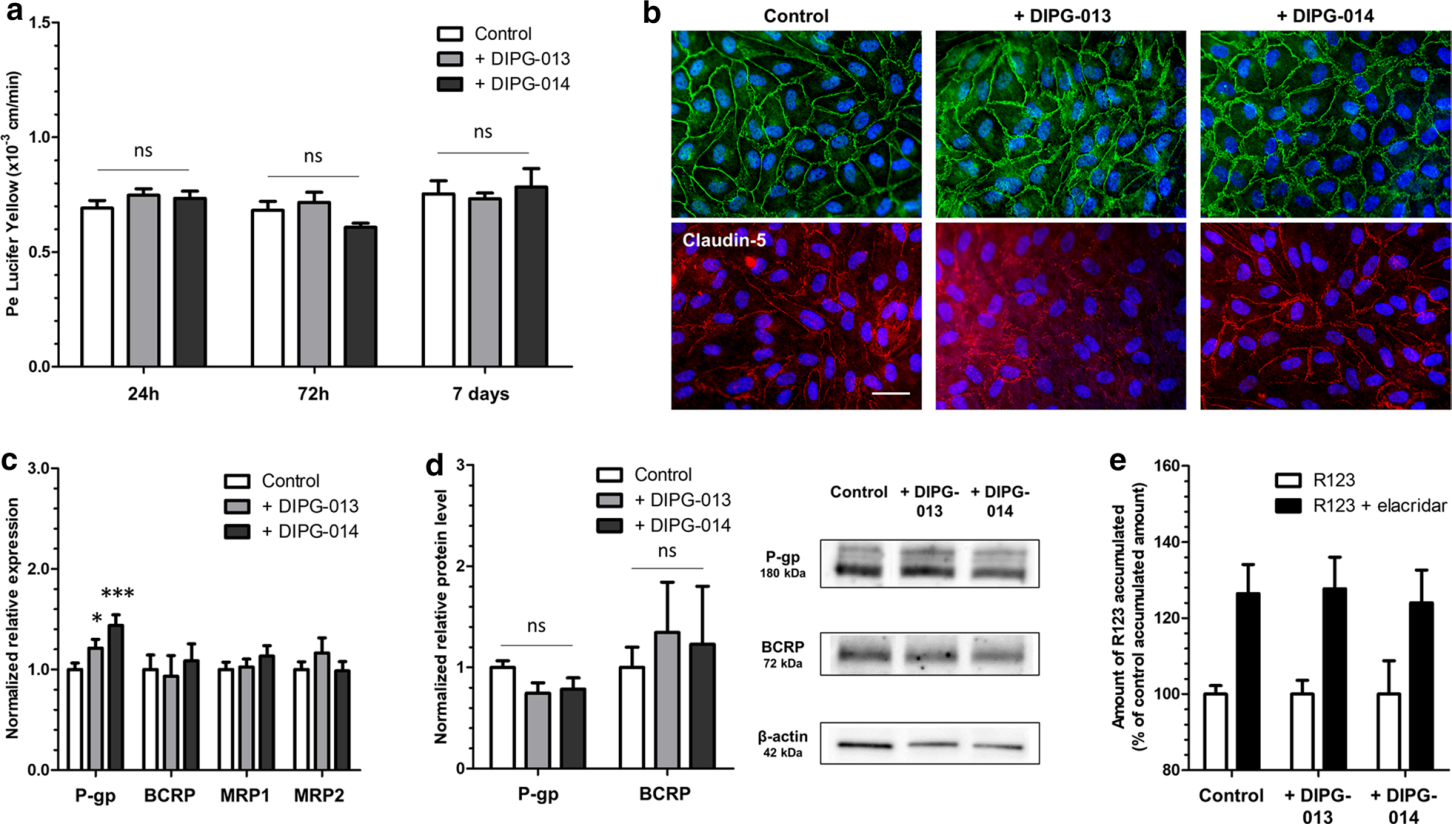
Impact of other DIPG cell lines on the physical and metabolic properties of the BBB. **a** Endothelial paracellular permeability (P*e*) to Lucifer Yellow after 24 h, 72 h and 7 days of incubation with astrocytes (control), DIPG-013 cells or DIPG-014 cells. Pe = endothelial permeability coefficient. N = 3; n = 9. ns = non significant. **b** Representative images of tight junction protein immunostainings of zonula occludens-1 (ZO-1) and claudin-5 after 7 days of incubation. Scale bar = 25 µm. **c** Transcriptional expression of the genes of efflux transporters P-gp, BCRP, MRP1 and MRP2, after 7 days of incubation, quantified by RT-qPCR and normalized on the expression of the housekeeping gene *PPIA* (cyclophilin A). ***p < 0.001; *p < 0.05. N = 2; n = 6. **d** Protein level of efflux transporters P-gp and BCRP, after 7 days of incubation, quantified by Western blot and normalized on the protein level of β-actin. Results were obtained from two independent experiments (N = 2). ns = non significant. **e** P-gp and BCRP activity, evaluated by the measure of P-gp/BCRP substrate rhodamine 123 (R123) accumulation in ECs, with or without the P-gp/BCRP inhibitor elacridar, after 7 days of incubation. N = 2; n = 6

According to the WHO classification, the HSJD-DIPG-013 (DIPG-013) and HSJD-DIPG-014 (DIPG-014) cell lines, expressing GFAP and presenting the same molecular profile as the DIPG-007, were used Table 1 (Additional file 1: Figure S1). In line with the results obtained with the DIPG-007 cell line, the physical integrity was maintained in the BBTB ECs with the DIPG-013 and DIPG-014 cells from 24 h (Pe^LY^ = 0.75 ± 0.03 × 10^−3^ cm/min and 0.73 ± 0.03 × 10^−3^ cm/min respectively *vs* control 0.69 ± 0.03 × 10^−3^ cm/min) until 7 days (0.73 ± 0.02 × 10^−3^ cm/min and 0.78 ± 0.08 × 10^−3^ cm/min respectively *vs* control 0.75 ± 0.05 × 10^−3^ cm/min) (Fig. 3a) and correlated with tight junction stainings (Fig. 3b).

Concerning the metabolic properties, while no significant modulation was measured for the expression of *ABCG2*, *ABCC1* and *ABCC2*, a significant increase in *ABCB1* gene expression was measured in the BBTB ECs with the DIPG-013 and DIPG-014 cells compared to BBB ECs (21% and 44% increase vs control respectively) (Fig. 3c). No significant increase in P-gp and BCRP protein levels was measured (Fig. 3d).

Anyhow, the same percentage of increase in accumulation was measured in the BBTB ECs with DIPG-013 and DIPG-014 cells  compared to the BBB ECs in presence of elacridar (28% and 24% respectively *vs* control 26%) revealing an absence of upregulation or downregulation of P-gp/BCRP activity (Fig. 3e). Hence, the increase in the efflux pump gene expression did not have an impact on the protein level and on the activity of the efflux pump itself.

The use of additional DIPG cells confirmed and validated the results obtained with the DIPG-007 cell line, such as the maintenance of the BBTB ECs integrity and the absence of regulation of efflux pumps at the protein and activity levels.

As the classical molecular protagonists usually suspected to be responsible for the chemoresistance phenotype did not seem to be involved, we then investigated the drug accessibility to the brain parenchyma in the BBTB models compare to the BBB model. To do so, the permeability of chemotherapeutic drugs across the BBB ECs and the BBTB ECs was measured.

### Chemotherapeutic drug transport across the BBB and BBTB models

**Fig. 4:**
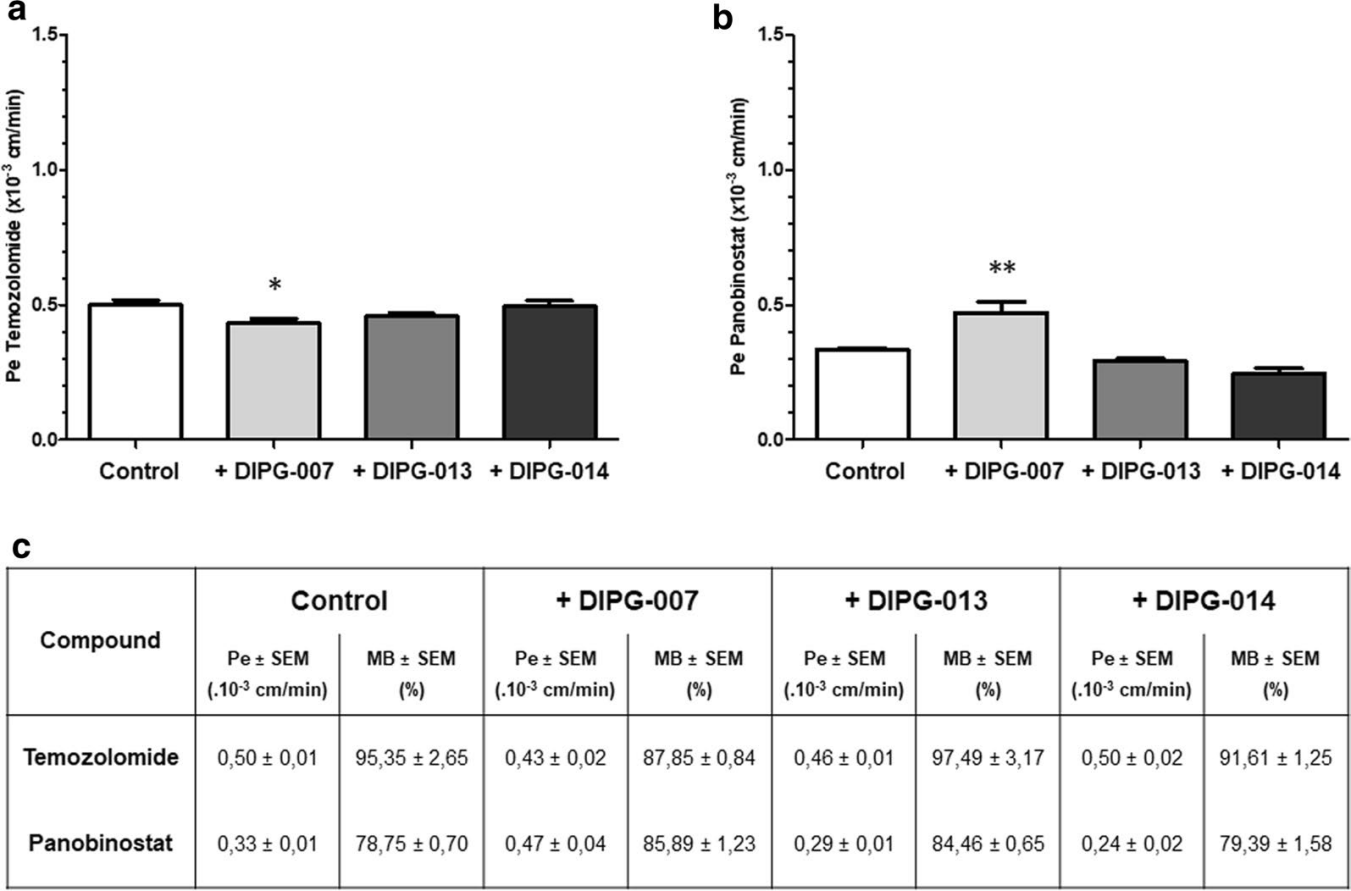
Impact of the DIPG environment on the transport of drugs across the BBB and BBTB. Endothelial permeability coefficient of the chemotherapeutic compounds **a** temozolomide and **b** panobinostat, after 7 days of incubation of the BBB cells with astrocytes (control), or DIPG cell lines DIPG-007, DIPG-013 and DIPG-014. **c** For each condition, the endothelial permeability coefficient (Pe) and the mass balance (MB) of the compounds were calculated. **p < 0.01; *p < 0.05. N = 2; n = 6

The endothelial permeability of selected chemotherapeutic compounds was measured through the BBTB ECs and compared to the BBB ECs. The selected compounds are temozolomide (TMZ) (Fig. 4a) as the gold standard and a targeted therapy compound, panobinostat (Fig. 4b).

For TMZ, while no significant modification of the Pe value was measured in the BBTB ECs with DIPG-013 and DIPG-014 cells (0.46 ± 0.01 × 10^−3^ cm/min and 0.50 ± 0.02 × 10^−3^ cm/min respectively) compared to the BBB ECs (0.50 ± 0.01 × 10^−3^ cm/min), the permeability was slightly lower in the BBTB ECs with DIPG-007 cells (0.43 ± 0.01 × 10^−3^ cm/min) (Fig. 4a–c).

At the opposite, the DIPG-007 environment induced an increase in permeability to panobinostat in the BBTB ECs compared to BBB ECs (0.47 ± 0.04 × 10^−3^ cm/min, 0.33 ± 0.01 × 10^−3^ cm/min respectively). No significant modification of the permeability coefficient was measured in the BBTB ECs with DIPG-013 and DIPG-014 (0.29 ± 0.01 × 10^−3^ cm/min, 0.24 ± 0.02 × 10^−3^ cm/min respectively) (Fig. 4b, c). For both compounds, the mass balance was included in a range between 78.75% until 97.49% (Fig. 4c), reflecting an absence of adsorption, degradation and uptake during the time of experiment, in line with the recommendation of Cecchelli et al. [25].

In summary, the permeability measurements of the chemotherapeutic drugs across the BBB ECs and BBTB ECs highlight that the DIPG environment does not restrict the transport of the drugs more than in the physiological condition.

### Are other metabolic properties involved in BBTB ECs chemoresistance?

**Fig. 5:**
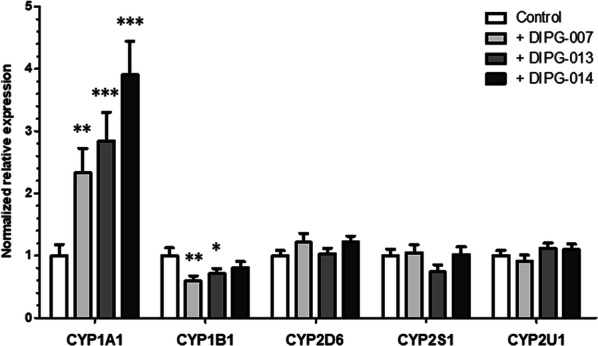
Detoxification enzymes expression in the BBB and the BBTB models. Transcriptional expression of the genes of cytochrome P450 enzymes *CYP1A1*, *CYP1B1*, *CYP2D6*, *CYP2S1* and *CYP2U1*, after 7 days of incubation, normalized on the expression of the housekeeping gene *PPIA* (cyclophilin A). ***p < 0.001; **p < 0.01; *p < 0.05. N = 3; n = 9

As no strong modulation of chemotherapeutic drug transport across the BBTB ECs vs the BBB ECs was measured, we wondered if other metabolic properties were modulated under the DIPG environment. To do so, the expression of Cytochrome P450 (CYP) enzymes was analyzed (Fig. 5). A significant increase in transcriptional expression was measured in the BBTB ECs for the gene encoding CYP1A1 with the DIPG-007, DIPG-013 and DIPG-014 cell lines (2.33; 2.84 and 3.91-fold increase vs. control respectively) (Fig. 5).

However, a 41% and 29% decrease in *CYP1B1* expression was measured in the BBTB ECs with DIPG-007 and DIPG-013 cells respectively, compared to the BBB ECs. No modulation of expression was detected for other CYP enzymes (*CYP2D6, CYP2S1, CYP2U1*) in all the conditions (Fig. 5).

To summarize, a significant increase in the expression of CYP1A1 and a decrease in the expression of CYP1B1 were detected in the BBTB ECs, suggesting an influence of the DIPG environment on the metabolism of BBTB ECs.

## Discussion

The BBB is a major hindrance to overcome for therapeutic compounds as it prevents their entry into the CNS and/or limits their access at a sufficient dose to achieve their therapeutic effect. In the last decade, even if a great endeavor was made to study the setting and the regulation of the BBB, the complexity of its architecture including the cellular interactions and the molecular mechanisms at play rendered the task intricate. In addition, these mechanisms can be dysregulated once the BBB is subjected to a pathological environment but the kinetic and properties are modulated according to each single pathology. In the field of cancer, and specifically for HGGs, it is even more true considering that molecular differences existing between adult and children HGGs clearly demonstrate that the results obtained from adult clinical trials cannot be simply extrapolated to children [6, 29]. This study is the first to develop a fully syngeneic human BBTB in vitro model specific to DIPG. The characterization of the physical and metabolic properties of the BBTB ECs showed the maintenance of the physical integrity as observed in clinic and revealed the absence of modulation of efflux pumps. The transport of the selected chemotherapeutic drugs across the BBTB ECs is not modified in the DIPG environment. However, the upregulation of expression of two CYP enzymes suggests an influence of the DIPG environment on the enzymatic component of the metabolic properties of the BBTB ECs.

The development of the BBTB model is based on our patented human BBB in vitro model [25] which had to be adapted to solely include human cells and to allow the culture of three cell types at the same time. The model was validated in terms of physical integrity and metabolic properties and possesses the required characteristics for a BBB in vitro model such as low permeability to a BBB integrity marker, a continuous localization of tight junction proteins and also functional efflux pumps [30].

When studied in vivo and depending on the method used, three to five weeks are necessary to obtain a DIPG model ready for molecular analysis or drug testing [7, 31–33]. In our in vitro model, the incubation with DIPG cells was done from 24 h to 7 days in order to create a chronic DIPG tumoral environment for the study of the BBTB ECs properties. The maintenance of the integrity measured in our model in the DIPG environment is in line with the clinical data, where no hyperdense signal is measured with Magnetic Resonance Imaging in children [22] contrary to what is observed in adult HGG [34–36].

The BBTB model was developed using three DIPG cell lines, chosen for their molecular profiles in accordance with the new classification of the WHO. The common molecular profile of the three cell lines enables a comparative approach in our study [2] and validates the reproducibility of our model.

The maintenance of the physical integrity of BBTB ECs excludes the access of drugs to the brain parenchyma through paracellular diffusion. Hence, drug delivery is dependent on the metabolic properties which restrict drug access to the brain parenchyma mainly through efflux transport and enzymatic detoxification. Among the efflux transporters, the members of the ABC family, P-gp, BCRP and MRPs, have a broad spectrum of substrates including anticancer drugs. Their expressions are often observed as being upregulated in the tumor vasculature, thus limiting even more drug access to the brain [16, 37].

These metabolic properties were then analyzed in the BBTB ECs and revealed that even if a slight increase in the transcriptional expression was measured for P-gp with the DIPG-013 and DIPG-014 cell lines, neither increase in the protein level nor in the activity of P-gp/BCRP were observed compared to the control model.

Thus, contrary to what is usually suspected and evocated, our result revealed an absence of a “super BBB" phenotype induced by the tumoral DIPG environment itself [34]. This result can be correlated with a recent study using an in vivo xenograft rat model of DIPG demonstrating an absence of modulation of efflux pumps [33]. However, only one study analyzed the expression of efflux pumps in the DIPG vasculature using histological analysis of pediatric DIPG brain tissue. Our results are also in accordance with these histological data revealing the expression of the efflux pumps P-gp, BCPR and MRP1 at the vasculature of DIPG patients in the vicinity of the tumor rather than in the tumor cells themselves. Nevertheless, our result concerning the absence of modifications of efflux pump expression observed in our BBTB model cannot be entirely comparable to this study, since with pediatric DIPG brain tissue resection, the authors were logically not able to study their expression in normal brainstem tissue but only in DIPG tissue [10]. In our in vitro study, no difference of efflux pump expression was observed in the BBTB model compared to the BBB model in which DIPG cells were replaced by brainstem astrocytes.

As the expression of efflux pumps is not modulated by the DIPG environment, we wondered if the accessibility to the brain parenchyma was restricted for chemotherapeutic drugs. Firstly, TMZ was chosen as one of the most effective drugs against brain tumors. Even if TMZ is described to be one of the few drugs to show a good permeability across the BBB, a low permeability to TMZ was measured in our system. While TMZ is described to be able to cross the BBB in non-human primate [38], the permeability coefficient across the BBB ECs measured in our human BBB in vitro model is equivalent to the BBB integrity marker. This basal permeability was comparable to the value obtained with another animal in vitro BBB model developed with porcine and rat cells [39]. However, the most important results concern the permeability value which was not modified in the BBTB ECs whatever the DIPG cells used, reflecting an absence of either increase or decrease in the TMZ permeability through the BBTB ECs. The tumoral environment does not seem to modify the access of TMZ to the brain parenchyma compared to the control condition. These results correlate with the absence of upregulation of the metabolic properties measured with the efflux pump expression and activity. However, we cannot exclude that in clinic, the access of TMZ to the brain parenchyma can be facilitated during the therapeutic sequence by its repeated and long term administration, since a down-regulation of the P-gp expression was measured in a human cell culture model after 72 h of treatment [40]. This effect could be even used in patients as a permeabilizing strategy to improve drug delivery if TMZ is administrated in combination with other drugs.

On the other hand, panobinostat, a pan-histone deacetylase inhibitor, is one of the most effective compounds against DIPG in pre-clinical models using in vitro and in vivo approaches [3, 41, 42]. However, the in vivo evaluation of its crossing capacity through the BBB seems to be dependent on the animal model used and results concerning the capacity of the compound to reach the tumor site are contested [41, 42]. Our in vitro data argue in favor of the results obtained in non-human primates [43] and in humans [44], and reveal a low BBB crossing of the compound. Moreover, even if a slight increase is measured through the BBTB ECs with the DIPG-007 cells, the permeability values remain low and equivalent to the BBB integrity marker. Hence, these results demonstrate the absence of reduction or increase in the crossing of the chemotherapeutic compounds through the BBTB ECs. The results are in line with the clinical data and validate the use of our human BBTB in vitro model as a tool for the prediction of brain penetration of drugs. However, as no biomarkers exist for DIPG and as endothelial permeability represents the only parameter that can be correlated with the clinical data, it would be interesting to analyze the molecular properties of brain capillaries isolated from DIPG vasculature to correlate our results.

Finally, even if the physical integrity and efflux pump activity of the BBTB ECs are not modulated by the DIPG environment, one cannot exclude that other metabolic properties can reduce brain accessibility, as suggested by the increase in expression of the detoxification enzyme CYP1A1. Indeed, drug biotransformation by CYP enzymes at the BBB ECs can contribute to CNS drug failure or toxicity, by conversion of drugs into inactive or harmful metabolites. Moreover, CYP enzymes can metabolize anticancer drugs, such as ifosfamide, vinblastine, etoposide and doxorubicine, and contribute to chemoresistance in patients [45]. Concerning TMZ and panobinostat, both drugs have been described as substrates of CYP enzymes [45, 46]. However, in our study, the mass balance values translate a nearly complete recovery of these therapeutic compounds, demonstrating an absence of adsorption, degradation and uptake in the BBB ECs and the BBTB ECs. The study of detoxification enzymes would require long-term exposure of ECs to drugs and the development of an effective enzymatic detection method, since no commercially available kits are sensitive enough to assess CYP activity in endothelial cells but are instead more adapted to hepatocytes.

Taking into account that chemotherapeutic drugs are able to modify the metabolic properties of the BBB, since TMZ was described to decrease P-gp expression [40] and panobinostat to inhibit CYP2D6 enzyme activity [46], the investigation of DIPG chemoresistance requires to adapt the study to mimic the therapeutic sequence including repeated dose exposure.

## Conclusion

In this study, we developed a human BBTB in vitro model specific to DIPG in order to evaluate the ECs properties in this specific pathological environment and to clarify the role of the BBTB in the chemoresistance observed in DIPG patients. The BBTB ECs do not display any increase in paracellular permeability, in line with clinical data. Moreover, contrary to what has been suggested, the BBTB ECs do not develop super properties compared to the BBB ECs. Indeed, even if the efflux pumps are active in the BBTB ECs, their typical increase in expression observed in the vasculature of adult HGGs does not seem to be a mechanism involved in DIPG chemoresistance. Although no in vitro BBB/BBTB models can fully represent the NVU, the relevance of the results concerning the permeability measurements of chemotherapeutic compounds in our human syngeneic model validates its use for the prediction of drug penetration to the brain.

## Supplementary information

**Additional file 1: Figure S1.** Morphological and molecular characterization of DIPG-013 and DIPG-014 cell lines.

## Data Availability

All relevant data are within the paper and its additional files.

## Abbreviations

BBB: Blood–brain barrier
BBTB: Blood–brain tumor barrier
BCRP: Breast cancer resistance protein
CNS: Central nervous system
CYP: Cytochrome P450
DIPG: Diffuse intrinsic pontine glioma
ECM: Endothelial cell medium
ECs: Endothelial cells
EGM: Endothelial cell growth medium
GEMMs: Genetically engineered mouse models
GFAP: Glial fibrillary acid protein
HGG: High grade glioma
LY: Lucifer yellow
MRP: Multi drug resistance protein
NGS: Normal goat serum
PBS-CMF: Phosphate buffer saline calcium magnesium free
PCR: Polymerase chain reaction
PDGFRβ: Platelet-derived growth factor receptor beta
Pe: Permeability coefficient
P-gp: P-glycoprotein
R123: Rhodamine 123
RH: Ringer Hepes
α-SMA: Alpha-smooth muscle actin
TMZ: Temozolomide
WHO: World Health Organization
ZO-1: Zonula occludens-1
