# Supplementary figures and images for: Development of a human in vitro blood–brain tumor barrier model of diffuse intrinsic pontine glioma to better understand the chemoresistance
Source: Fluids Barriers CNS. 2020 Jun 2;17:37. doi: 10.1186/s12987-020-00198-0 (PMC7268424; doi:10.1186/s12987-020-00198-0)

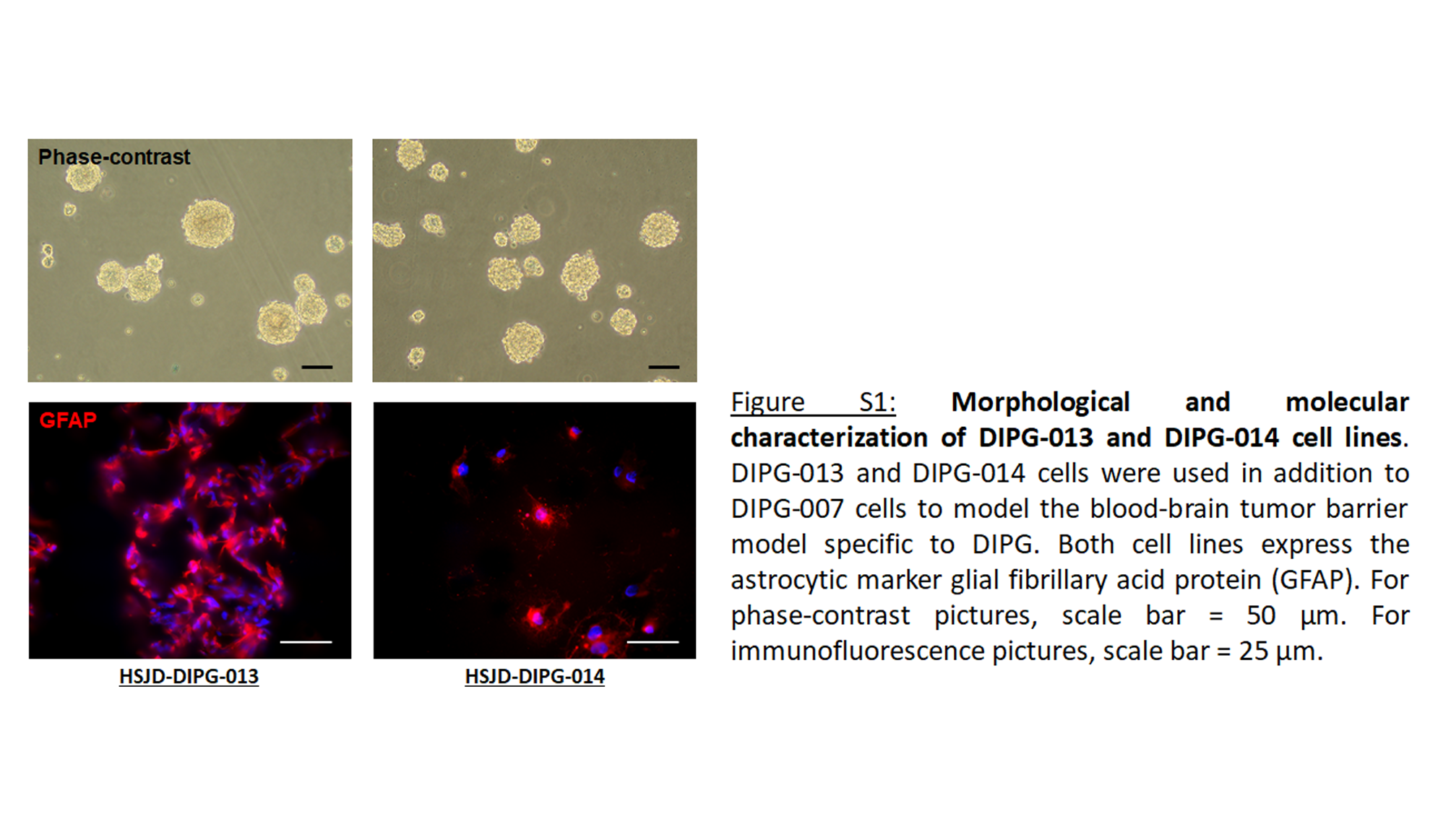

Supplement: Supplementary file 1 — Additional file 1: Figure S1. Morphological and molecular characterization of DIPG-013 and DIPG-014 cell lines. [file 12987_2020_198_MOESM1_ESM.tif]
